# The use of integrated text mining and protein-protein interaction approach to evaluate the effects of combined chemotherapeutic and chemopreventive agents in cancer therapy

**DOI:** 10.1371/journal.pone.0276458

**Published:** 2022-11-11

**Authors:** Mohammad Rasoul Samandari Bahraseman, Babak Khorsand, Keyvan Esmaeilzadeh-Salestani, Solmaz Sarhadi, Nima Hatami, Banafsheh Khaleghdoust, Evelin Loit

**Affiliations:** 1 Faculty of Sciences, Department of Biology, Shahid Bahonar University of Kerman, Kerman, Iran; 2 Varjavand Kesht Kariman, Limited Liability Company, Kerman, Iran; 3 Computer Engineering Department, Ferdowsi University of Mashhad, Mashhad, Iran; 4 Chair of Crop Science and Plant Biology, Institute of Agricultural and Environmental Sciences, Estonian University of Life Sciences, Kreutzwaldi, Tartu, Estonia; 5 Kerman Department of Endodontic Dentistry, Kerman University of Medical Sciences, Kerman, Iran; University of Agriculture in Krakow, POLAND

## Abstract

Combining chemotherapeutic (CT) and chemopreventive (CP) agents for cancer treatment is controversial, and the issue has not yet been conclusively resolved. In this study, by integrating text mining and protein-protein interaction (PPI), the combined effects of these two kinds of agents in cancer treatment were investigated. First, text mining was performed by the Pathway Studio database to study the effects of various agents (CP and CT) on cancer-related processes. Then, each group’s most important hub genes were obtained by calculating different centralities. Finally, the results of *in silico* analysis were validated by examining the combined effects of hesperetin (Hst) and vincristine (VCR) on MCF-7 cells. In general, the results of the in silico analysis revealed that the combination of these two kinds of agents could be useful for treating cancer. However, the PPI analysis revealed that there were a few important proteins that could be targeted for intelligent therapy while giving treatment with these agents. *In vitro* experiments confirmed the results of the *in silico* analysis. Also, Hst and VCR had good harmony in modulating the hub genes obtained from the *in silico* analysis and inducing apoptosis in the MCF-7 cell line.

## Introduction

Cancer is one of the leading causes of death in the world. If cells damaged by mutations cannot repair themselves and inhibit cell growth during the tumorigenic process, they will become cancer cells [1]. The most common treatments against cancer include chemotherapy, surgery, and radiation therapy, each of which has its own disadvantages [2]. For example, chemotherapy as a conventional method leads to unwanted side effects and drug resistance [3,4]. Therefore, finding new therapeutic strategies is one of the most important concerns of researchers who are trying to improve the cancer treatment processes.

The term chemoprevention, which refers to inhibiting, suppressing, or reversing cancer progression by using natural or synthetic compounds, was first coined by Sporn in the early 1970s [5]. Introducing an agent as a chemopreventive (CP) agent, with no side effects is one of the prerequisites [6]. In addition to their preventive properties, CP agents are effective in inhibiting cell proliferation and inducing apoptosis in human cancer cells [7]; thus, it can be concluded that these agents can play important therapeutic roles in cancer treatment.

One of the most essential strategies for improving the effectiveness of different drugs used for cancer treatment is combination therapy. The synergistic effect could increase the performance of various anti-cancer drugs [8–11]. Numerous investigations have indicated that CP agents, in combination with CT, can induce an additive or synergistic effect in stimulating cell death. Nevertheless, the application of these agents for cancer therapy or in combination therapy with CT agents to improve treatment efficiency is controversial and has not reached a definitive conclusion due to insufficient information [12,13]. For example, some clinical studies have reported that the application of CP agents in combination with CT agents was ineffective for cancer therapy and can lead to an increased risk of death in some cases [14,15]. Therefore, scrutinizing the combined effects of these factors in cancer therapy is crucial.

Vincristine (VCR) is a natural vinca alkaloid derived from the plant *Catharanthus roseus* and is widely used as a CT agent in treating various cancers [16,17]. Although VCR has a long history of being used in the fight against cancer, its dose-dependent side effects cause neurotoxicity [18,19]. Hesperetin (Hst) is a flavonoid subgroup found naturally in citrus fruits [20]. Numerous preclinical studies have shown that Hst has a protective role against cancer’s malignant progression through various cellular signaling pathways [21,22]. So far, no studies have been conducted on the combined effects of these two factors on cancer.

Pathway Studio is a comprehensive database of full-text articles, abstracts, and clinical trial information, covering more than 10,000 journals and giving users access to the largest biomedical database and related content. By providing statistical tools, this database allows researchers to extract and analyze their experimental and applied information [23].

Since we aimed at investigating whether CP agents could be effective in adjunctive therapy in cancer treatment when used along with CT agents, five CP agents were selected so their effects could be evaluated when used in combination with two CT agents. This study presents a pipeline in which, by evaluating the effects of these agents on the proteins involved in cancer-related signaling pathways, the negative or positive effects of these two types of agents on cancer treatment could be determined. The effect of the combination of VCR and Hst was measured against MCF-7 cancer cells to validate the *in silico* results. Researchers can use this method in the future to investigate the combined effect of various drugs on diseases.

## Materials and methods

### Text-mining analysis

The effects of CP and CT agents on cellular signaling pathways related to cancer were evaluated using the Pathway Studio Mammal Plus database [23]. Five CP (curcumin, resveratrol, quercetin, silibinin, and Hst) and two CT agents (doxorubicin and VCR) were selected as an input, and various cancer-related cellular signaling processes were selected as the targets to be analyzed by the Pathway Studio Mammal Plus software in 2020 (www.pathwaystudio.com). The pathway-studio output contains information about the impact of each input on the target as a "positive effect," "negative effect," or "both positive and negative effects." First, the obtained information was carefully reviewed to evaluate the accuracy of the mined text. Although the results of the Pathway Studio were sometimes not correct, overall, they were accurate in over 95 percent of the cases. Then, the results were categorized into four "positive," "negative," "unknown," and “dual-role” groups. The logic behind this classification was based on the effects of each agent on the cancer treatment process. For example, where the effects of the agents on the P53 were positive in the Pathway Studio results, it was considered a positive effect in the treatment of cancer. The results of most findings in the various literature were considered a decision criterion to determine genes that have a dual role (positive and negative) in the treatment processes. For example, genes such as NF-κB are involved in different processes, and many studies have reported that increased expression of this gene causes resistance to cancer treatment, but some studies have also shown the effectiveness of this gene in inducing apoptosis. Moreover, the increase of intracellular ROS was considered a stimulus for cell death, so the effect of genes related to this process was classified accordingly (S1 Data).

### Enrichment and network analysis

The text-mining results were divided into four groups based on their effects on cancer treatment for network analysis. Group 1: The effects of both CT and CP agents on cancer treatment were positive. Group 2: The effects of CP agents on the treatment process were positive, and the effects of CT agents were negative. Group 3: The effects of CT agents were positive, and the effects of CP agents were negative. Group 4: The effects of both CT and CP agents were negative.

Human protein-protein interaction network (HPPIN) is a graph in which the nodes are human proteins (HPs), and the edges show the interaction between them (S1 Fig). The HPPIN has 288989 experimental interactions between 20748 HPs [24]. Different centralities are used to score each HP [25].

Degree (connectivity): Degree centrality of per HP (*D*_*i*_ for *HP*_*i*_) is the digit of its neighbors in HPPIN.

Neighborhood connectivity: Neighborhood connectivity per HP (*N*_*i*_ for *HP*_*i*_) is the average degree of its neighbors which is described as *Eq 1*.


$$N_{i}=\sum_{j\in D_{i}}D_{j}$$ (1)

Shortest paths: The shortest path centrality (*S*_*i*_ for *HP*_*i*_), as it is illustrated in *Eq 2*, is the synopsis of the shortest path between that HP and all the other HPs divided by the entire number of HPs (*n*). The shortest path between nodes *i* and *j* (*S*_*i*,*j*_) is the number of edges that should be traversed to reach *j* from *i*.


$$S_{i}=(\sum_{j=1}^{n}S_{i,j})/n$$ (2)

Betweenness centrality: The betweenness centrality of each HP is characterized as the ratio of the shortest paths that pass via that HP which is represented as *Eq 3*. *σ*_*j*,*k*_ and it is the number of the shortest path between *j* and *k*, while *σ*_*j*,*k*_(*i*) is the number of the shortest paths between *j* and *k* which pass through *i*.


$$B_{i}=\sum_{j=1}^{n-1}\sum_{k=j}^{n}\sigma_{j,k}(i)/\sigma_{j,k})$$ (3)

Closeness centrality: The closeness centrality of an HP represents the joint of its average shortest path distance in the whole HPPIN.

Diversity of Predators: In the Human-Virus PPI network, for each HP, each of the virus families that interact with it is called a predator. For each HP, the mean evolutionary length of its predators multiplied by the number of its predators is regarded as its diversity of predators (DP) score [26].

The igraph package calculated the centrality measures in the R programming language. All these centralities were calculated in each group, and the genes with the highest scores were selected as hub genes. For groups 1 and 2, which had the largest number of genes, the evaluation was done on the top 10 centralities, while for the next groups, which had fewer genes, the top 5 centralities were evaluated. Finally, enrichment analysis was performed by the DAVID database to investigate the effects of these hub genes on the biological processes.

### Cell culture

MCF-7 cells (human breast adenocarcinoma cell line) were purchased from the Iranian Genetic Resources Center. The MCF-7 cells were grown in DMEM medium with 10% fetal bovine serum and the antibiotics penicillin (100 U/ml) and streptomycin (100 g/ml). The cells were cultured at a density of 5000 cells per well in a 96-well plate for MTT testing. For western blotting and real-time PCR, MCF-7 cells were grown in a 6-well plate and allowed to bind and grow for 24 h for the protein and RNA to be extracted. Then, the cells were treated with VCR, Hst, and the combined dose and were also incubated for 24 h.

### Cell viability analysis

Cell viability was evaluated using the MTT assay, which is based on the reduction of MTT (3- (4,5-dimethylthiazol-2-yl)-2,5-diphenyltetrazolium bromide) by a mitochondrial dehydrogenase into an aqueous formulation product. The MTT powder was dissolved in PBS with a final concentration of 0.5 μg/ml. After treatment of the cells in the 96-well plate, they were incubated for 24 h. Then, 30 μL of dissolved MTT was added to each well, and the wells were kept in an incubator for 3 h. After removal of the medium culture, 100 μL of DMSO was added to each well to dissolve the formed formazan crystals. Finally, the amount of formazan was measured by measuring the absorbance at 490 nm by an ELISA plate reader (Elisa MAT 2000, DRG Instruments, GmbH, Marburg, Germany).

### Immunoblot analysis

For western blotting, firstly, MCF-7 cells were seeded on a six-well plate and allowed to attach for 24 h. Then, the cells were treated with the intended concentrations of VCR, Hst, and their combination and were also incubated for 24 h. After this, the cells were homogenized by centrifugation at 4°C at 14000 rpm for 14 min with a buffer containing EDTA (1 mM), Na-deoxycholate (0.1%), SDS (0.1%)‌, NP-40 (1%), Tris-HCl (10 mM), protease inhibitors, and sodium orthovanadate (1 mM). The resulting supernatant was drawn and maintained. Protein concentration was measured using the Bradford technique (Bio-Rad Laboratories, Germany). Adequate quantities of protein were injected into a gel with 9% SDS-PAGE, which was then transferred to the nitrocellulose membrane by electrophoresis (Hybond ECL, GE Healthcare Bio-Sciences, USA). After an overnight blockage at 4°C with 5% skim milk powder in tris-buffer solution and Tween 20 (blocking buffer, TBS-T, 150 mM NaCl, 20 mM Tris-HCl, pH 7.5, 0.1% Tween20), the membrane was impregnated with caspase-3 and NF-κB (p65) (Cell Signaling Technology, Danvers, MA, USA, overnight at 4°C) at room temperature. After washing with TBS-T (three times, 5 min each time), the stains were incubated for 60 min at room temperature with a secondary peroxidase antibody (1: 15000, GE Healthcare Bio-Sciences Corp. NJ, USA). All antibodies were diluted in the blocking buffer. The antibody-antigen complexes were identified using the ECL system and exposed to the Lumi-Film chemical detection film (Roche Applied Science, Mannheim, Germany). Image j software was employed to estimate the expression intensity. The GAPDH (Cell Signaling Technology, INC. Beverly, MA, USA; 1: 1000) was used as load control.

### RNA extraction and real-time PCR

After the 24-hour treatment of the cells with Hst (120 μM), VCR (200 nM), and their combined dose, the RNA was isolated using an RNA extraction kit (AsiaTech Kit, Iran) according to the manufacturer’s instructions. A NanoDrop™ 2000/2000c spectrophotometer was utilized to calculate the quality and concentration of the extracted RNA. Then, the cDNA was synthesized by the Thermo Scientific™ Fermentas kit. After cDNA synthesis, real-time PCR was done using the RealQ Plus 2x Master Mix (AMPLIQON). The primers designed by Oligo 7 software were controlled for their specificity using BLAST on the NCBI site. The formula 2^-ΔΔCT^ was used to estimate the percentage of the relative gene expression. The *GAPDH* gene was used as a housekeeping gene. The primer sequences are shown in Table 1.

**Table 1:** Primers sequences used in the study.

Gene	Forward primer	Reverse primer
***NF-κB***	5’TGATGATTTACTAGCACAAGG3’	5’ ATTATTAAGTATCCCCAGACC 3’
***MYC***	5’ CCCAAACCAGAAATGATGTTG3’	5’ GACCTACTTTGAGACTGAGAC 3’
***GAPDH***	5’CCCCAGCAAGAGCACAAGAGG3’	5’ AGGAGGGGAGATTCAGTGTGG 3’
***BCL-2***	5’ TGGGGTCATGTGTGTGGAG3’	5’ CGGTTCAGGTACTCAGTCATCC 3’
***P53***	5’ ACCTAAAAGGAAATCTCACCC3’	5’ ACCCTGAGCATAAAACAAGTC 3’
***JUN***	5’CTTGAAAGCTCAGAACTCGGAG3’	5’ TGCTGCGTTAGCATGAGTTGGC3’

### Statistical analysis

The data were analyzed using ANOVA. All experiments were done with at least three replications. The western blot band density was measured using Image j software. The results are expressed as mean ± SEM. Statistical significance was defined as *P* < 0.05, *P* < 0.01, and *P* < 0.001.

## Results

### *In silico* analyses

#### Evaluation of the effects of agents on cancer-related cellular pathways by text mining

**Fig 1:**
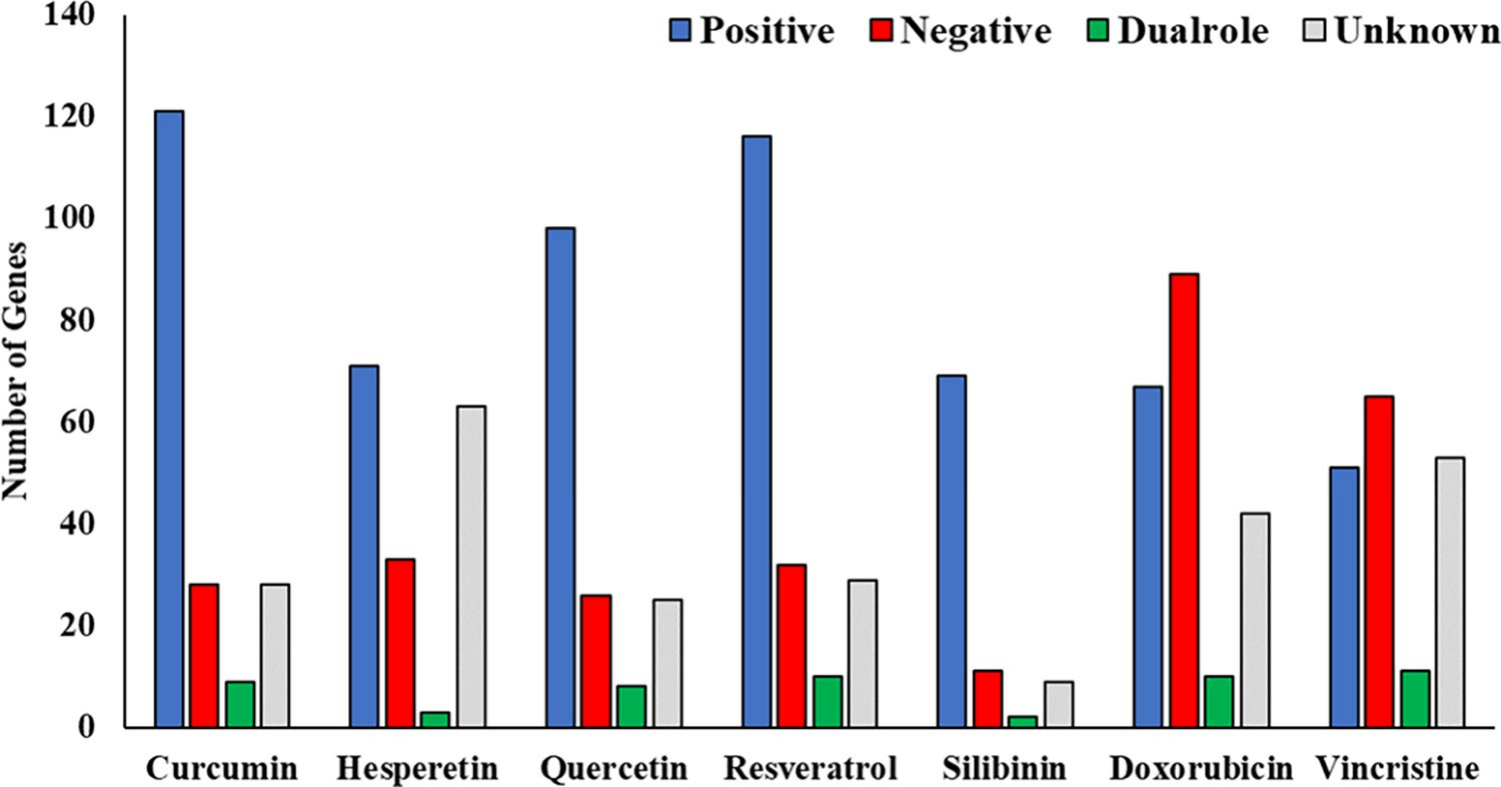
The effects of each agent on cellular signaling genes involved in cancer. Blue: Positive effect on cancer treatment. Red: Negative effect on cancer treatment. Gray: Not available in the literature. Green: Dual-role effect on cancer treatment.

**Fig 2:**
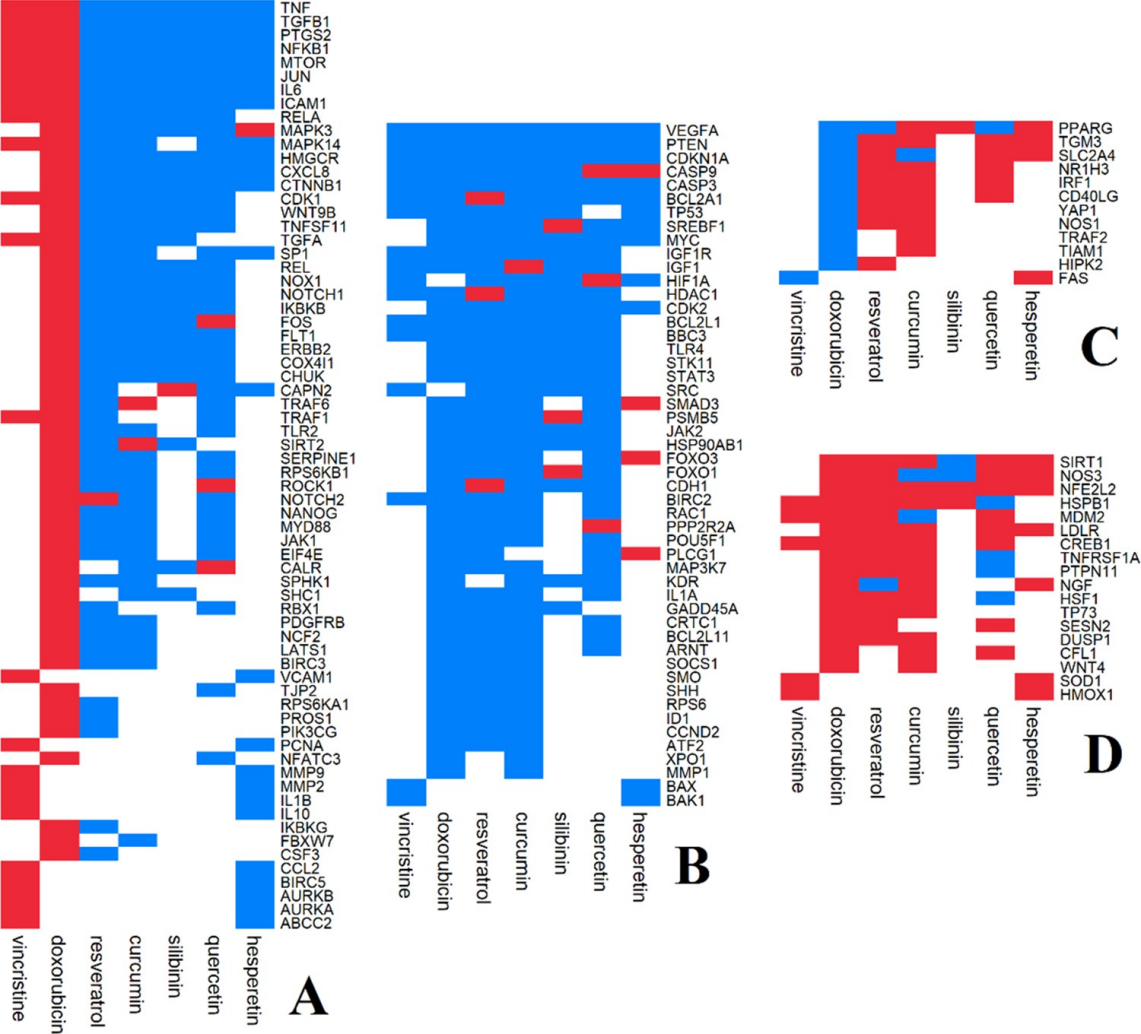
The effects of agent efficacy on the cancer treatment process through text-mining analysis. A: Group 2 indicated the positive effects of CP and adverse effects of CT on cancer treatment. B: Group 1 indicated that both agents had positive effects on cancer treatment. C: Group 3 indicated that CT had positive effects on cancer treatment, but CP had negative effects on cancer treatment. D: Group 4 indicated that both CT and CP had negative effects on cancer treatment. Blue: Positive effect on cancer treatment. Red: Negative effect on cancer treatment. White: Not available in the literature.

The results indicated that CT and CP agents had different impacts on cancer treatment. Clustering the results revealed that the effects of the two types of agents on the genes were different, so the two CT agents were placed in one cluster, and the five CP agents were placed in the other cluster (S2 Fig). Among all the studied agents, curcumin and doxorubicin showed the minimum and maximum disadvantages for cancer therapy, respectively. An overview of the results illustrated that CT agents had more negative effects on cancer treatment than CP agents (Fig 1). According to the grouping analysis, Group 2 and Group 1 had the highest number of genes (Fig 2A and 2B), while Group 3 showed the lowest number of genes (Fig 2D).

#### Protein-protein interaction network analysis

**Fig 3:**
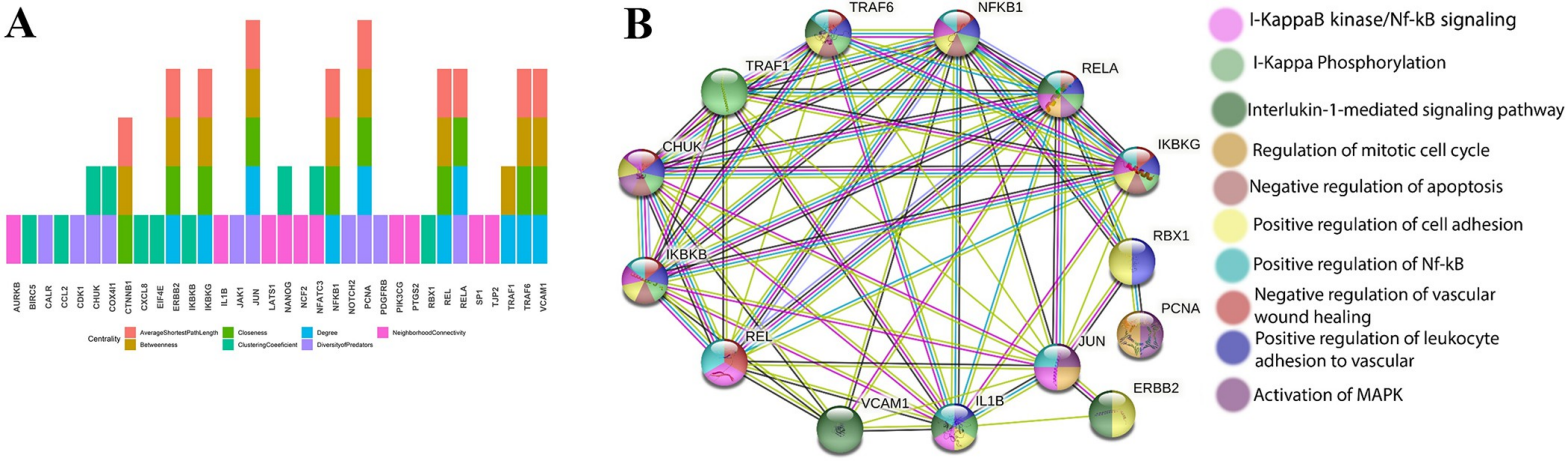
PPI analysis. A: Hub genes of the top 10 centralities obtained from PPI analysis (Group 2). B: The PPI of the most scored hub genes of the top 10 centralities (Group 2) were obtained through the string database (https://string-db.org/).

The most important hub genes of each group were identified by examining the PPI network’s six centralities. In Group 1, the genes P53, SRC, RAC1, RPS6, MYC, HSP90AB1, HDAC1, and CDKNA1 (S3 Fig) had the highest score among different centralities. The genes JUN, NF-κB, PCNA, REL, RELA, TRAF, and VCAM1 were the most important hub genes in Group 2 (Fig 3A). The genes YAP1, TRAF2, YAP, TIAM1, and PPARG in Group 3, and genes CLF1, HSPB1, MDM2, SIRT1, and SOD1 (S3 Fig) in Group 4 were the most influential hub genes.

The PPI of Group 2 hub genes indicated that the hub genes of this group were highly correlated with each other and formed a very close unity. The most important pathways associated with these proteins were NF-κB activity, cell adhesion, vascular wound healing, cell cycle, and apoptosis (Fig 3B).

#### Enrichment analysis

The enrichment analysis of hub genes showed that different groups were involved in many cancer-related processes. Twenty-four terms were significant in the KEGG pathway (KP) analysis, and 120 terms were significant in biological processes (BPs) for Group 1. Most of the KPs were related to cancer, and most of the BPs were related to apoptosis and cell growth (S2 Data). In Group 2, 60 terms were significant in the KPs, and 104 terms were significant in the BPs. The most important processes related to this group are summarized in Tables 2 and 3. The NF-κB signaling pathway was significant in both databases, and other processes were strongly associated with cancer. In Group 3, three terms were significant in the KPs, and 25 were significant in the BPs, among which the processes related to apoptosis were the most common. In Group 4, three terms in the KPs and 54 terms in the BPs were significant, of which the processes related to cellular redox were the most common (S2 Data).

**Table 2:** Pathways enriched by KEGG for hub genes of Group 2.

Term	%	P-Value	Genes
**hsa04668: TNF signaling pathway**	35.2	7.5E-13	IKBKB, JUN, VCAM1, CHUK, IL1B, CCL2, TRAF1, IKBKG, PTGS2, RELA, NFKB1, PIK3CG
**hsa05200: Pathways in cancer**	50.0	2.4E-12	PDGFRB, JUN, CXCL8, CHUK, TRAF1, PTGS2, PIK3CG, RELA, NFKB1, RBX1, IKBKB, TRAF6, ERBB2, BIRC5, CTNNB1, IKBKG, JAK1
**hsa04064: NF-κB signaling pathway**	32.3	3.1E-12	IKBKB, VCAM1, CXCL8, CHUK, IL1B, TRAF6, TRAF1, IKBKG, PTGS2, RELA, NFKB1
**hsa05222: Small cell lung cancer**	26.4	3.2E-9	IKBKB, CHUK, TRAF6, TRAF1, IKBKG, PTGS2, RELA, NFKB1, PIK3CG
**hsa05215: Prostate cancer**	26.4	4.2E-9	PDGFRB, IKBKB, CHUK, ERBB2, CTNNB1, IKBKG, RELA, NFKB1, PIK3CG
**hsa05212: Pancreatic cancer**	23.5	1.3E-8	IKBKB, CHUK, ERBB2, IKBKG, RELA, NFKB1, PIK3CG, JAK1
**hsa04010: MAPK signaling pathway**	29.4	1.4E-6	PDGFRB, IKBKB, JUN, CHUK, IL1B, TRAF6, NFATC3, IKBKG, RELA, NFKB1
**hsa04210: Apoptosis**	17.6	8.4E-6	IKBKB, CHUK, IKBKG, RELA, NFKB1, PIK3CG
**hsa04014: Ras signaling pathway**	23.5	6.2E-5	PDGFRB, IKBKB, CHUK, REL, IKBKG, RELA, NFKB1, PIK3CG
**hsa04151: PI3K-Akt signaling pathway**	26.4	1.3E-4	PDGFRB, IKBKB, CHUK, IKBKG, EIF4E, RELA, NFKB1, PIK3CG, JAK1

**Table 3:** Biological possesses enriched by DAVID for hub genes of Group 2.

Term	%	P-Value	Genes
**GO:0006954~inflammatory response**	35.2	8.3E-11	IKBKB, CXCL8, CHUK, IL1B, NFATC3, REL, CCL2, IKBKG, PTGS2, RELA, NFKB1, PIK3CG
**GO:0051092~positive regulation of NF-κB transcription factor activity**	23.5	6.0E-9	IKBKB, CHUK, IL1B, TRAF6, TRAF1, IKBKG, RELA, NFKB1
**GO:0071356~cellular response to tumor necrosis factor**	17.6	2.2E-6	IKBKB, VCAM1, CXCL8, CHUK, CCL2, RELA
**GO:0051403~stress-activated MAPK cascade**	11.7	8.9E-6	IKBKB, CHUK, IKBKG, NFKB1
**GO:0010803~regulation of tumor necrosis factor-mediated signaling pathway**	11.7	2.7E-5	IKBKB, CHUK, TRAF1, IKBKG
**GO:0006915~apoptotic process**	20.5	7.3E-4	NOTCH2, IL1B, CDK1, BIRC5, TRAF1, IKBKG, NFKB1
**GO:0001525~angiogenesis**	14.7	9.1E-4	JUN, CXCL8, CCL2, PTGS2, PIK3CG
**GO:0000082~G1/S transition of mitotic cell cycle**	11.7	0.001	LATS1, PCNA, CDK1, EIF4E

### *In vitro* validation of data

#### Overview of integrated text mining and PPI for the combination of Hst and VCR

**Fig 4:**
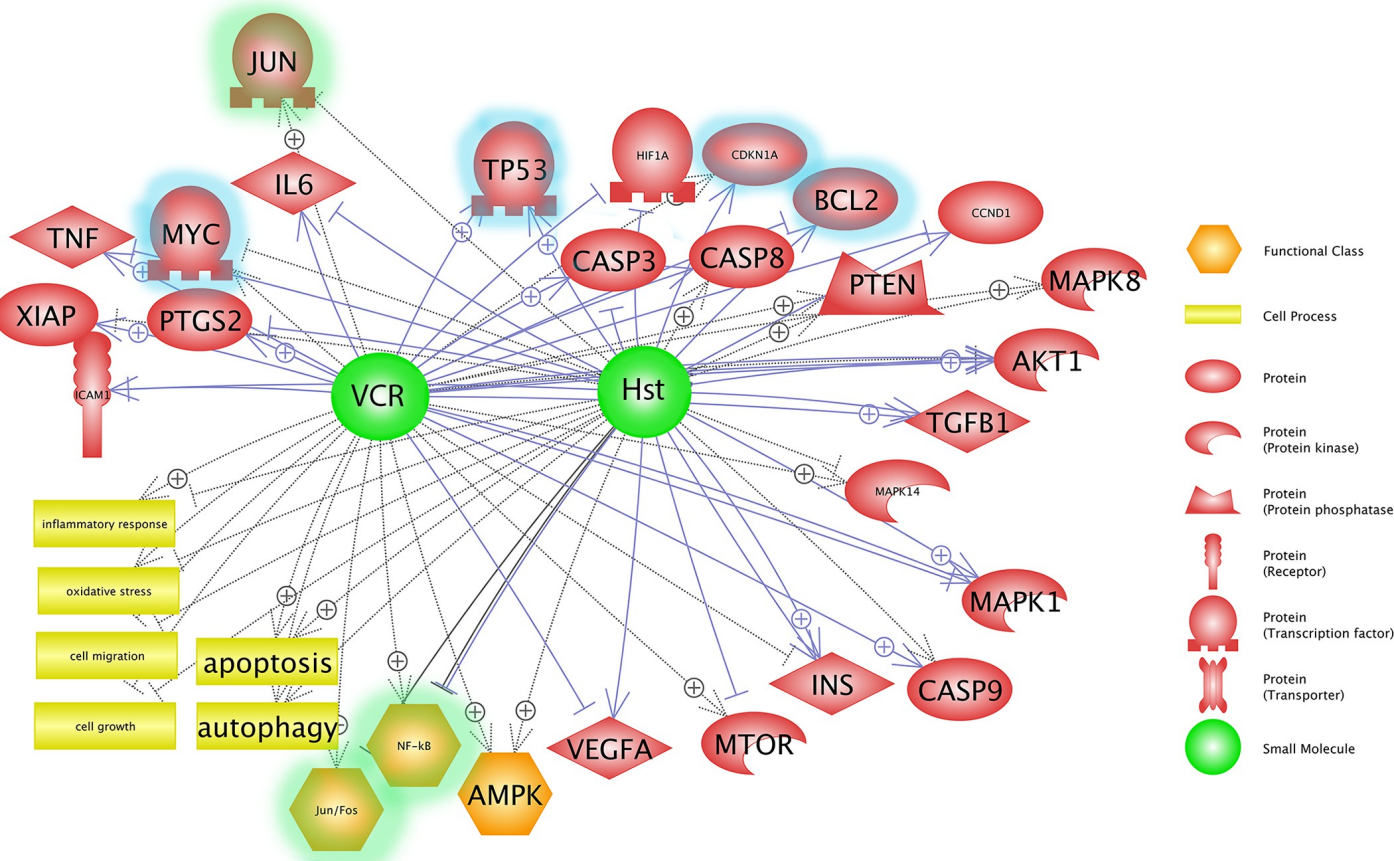
Integrated text mining and PPI for the combined effects of Hst and VCR on cancer-related signaling pathways. The graph was obtained from pathway studio software, and the highlighted colors were added based on PPI analysis. Green highlight: Hub genes of Group 1. Blue highlight: Hub genes of Group 2.

Fig 4 shows the integration of text mining and PPI for the two agents, VCR and Hst, selected for the practical tests. The hub genes obtained from PPI analysis are highlighted in this graph. The hub genes obtained from the PPI network analysis were selected to be examined against the MCF-7 cancer cell line to validate *in silico* analysis.

#### The effects of VCR and Hst on MCF-7 cell viability

**Fig 5:**
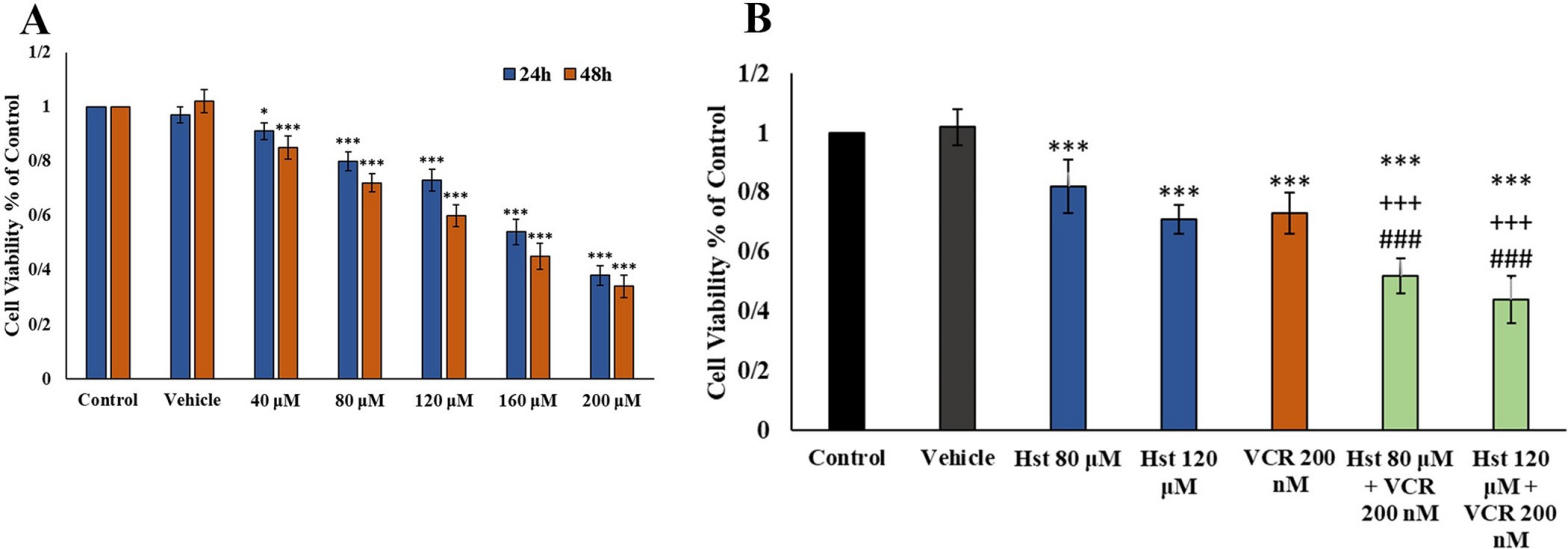
The cell viability assay. (A) The effects of Hst on MCF-7 cell viability after 24 h and 48 h. (B) The effects of combined doses of Hst and VCR on MCF-7 after 24 h. *: 0.05, ***: 0.001 indicates a significant level of control. The level of significance of the combined doses was calculated compared to the corresponding doses in the non-combined state. ###: 0.001 indicates a significant degree of combined groups compared with the VCR group. +++: 0.001 indicates a significant degree of the combined group compared with the related Hst group.

The MTT assay indicated that Hst could reduce cell viability in a time- and dose-dependent manner in the MCF-7 cell line (Fig 5A). The IC_50_ of Hst was approximately 160 μM for 24 h. VCR at a dose of 400 nM had an IC_50_ (The results are not reported). The combination doses did not have a synergistic effect, and their effect on cell viability was mainly cumulative (Fig 5B). The doses of 120 μM Hst, 200 nM VCR, and their combination were selected for molecular experiments.

#### Evaluation of hub genes derived from *in silico* analysis

**Fig 6:**
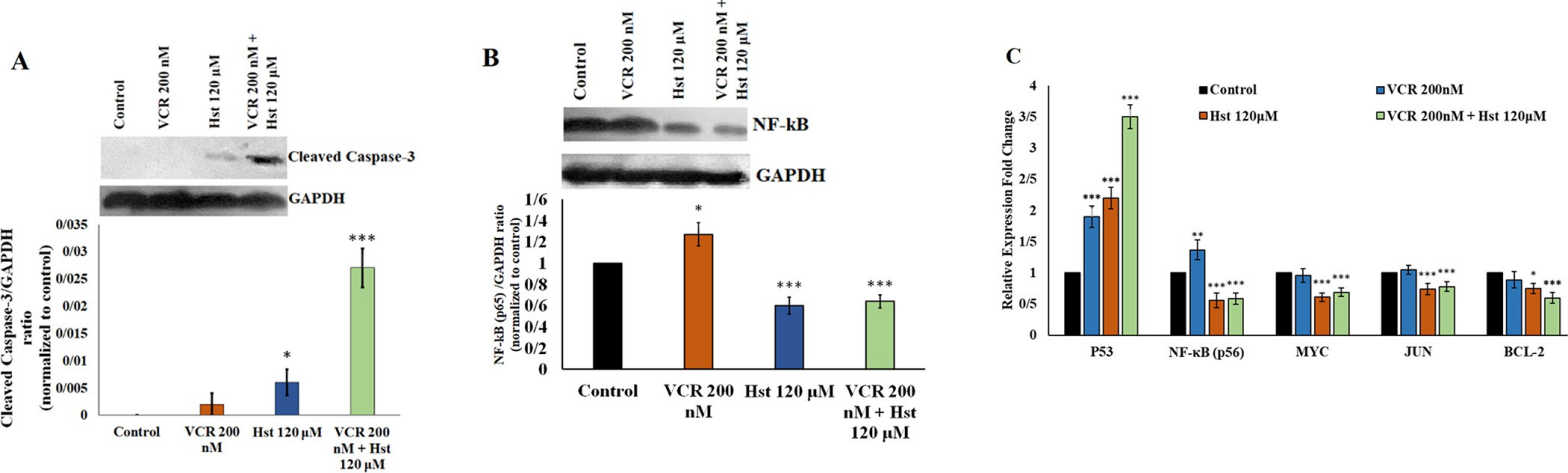
The effects of Hst, VCR and the combination dose on the expression of hub genes were obtained from *in silico* analysis in MCF-7 cells. (A): Western blot of cleaved caspase-3 protein after treatment with 120 μM Hst, 200 nM VCR, and the combination of VCR and Hst after 24 h in the MCF-7 cell line. (B): Western blot of NF-κB protein after treatment with 120 μM Hst, 200 nM VCR, and the combination of VCR and Hst after 24 h in the MCF-7 cell line. (C): Relative expression analysis of hub genes by real-time PCR after treatment with 120 μM Hst, 200 nM VCR, and the combination of Hst and VCR for 24 h. *: 0.05, **: 0.01, and ***: 0.001, show a significant level of control.

The combination of Hst and VCR induced apoptosis in the MCF-7 cell line. The combined group was significantly able to induce caspase-3 cleavage. However, the caspase-3 cleavage was much lower in the Hst group (120 μM) compared to the combined group, and no caspase-3 cleavage was observed at 200 nM VCR (Fig 6A).

The results showed that Hst significantly reduced the expression of NF-κB protein. Treatment of MCF-7 with 200 nM VCR increased the expression of this protein. The combination of Hst and VCR significantly decreased the amount of NF-κB protein compared to the control group (Fig 6B).

The other hub genes derived from *in silico* analysis were measured by real-time PCR. Hst and VCR significantly increased the expression of *TP53*. The elevated expression level of this gene was significantly higher in the combined dose compared to the application of each agent individually (Fig 6C). The 120 μM Hst dose and the combined dose reduced the *MYC* gene expression. However, 200 nM VCR did not alter the expression of this gene. There was a significant decrease in the expression of *JUN* treated by Hst. The expression of *JUN* did not show a significant difference under treatment with VCR, while the combined dose of Hst and VCR significantly reduced the expression of this gene. Treatment with Hst, VCR, and the combination dose, significantly reduced the expression of the *BCL-2* gene in MCF-7 cells (Fig 6C). The results of real-time PCR for *NF-κB* were similar to those of western blotting, described above.

## Discussion

Many studies have been conducted to evaluate the preventive effects of CP agents against cancer [27–29]. Although CPs with low or no side effects are an interesting choice for combination cancer therapy, the benefit of these agents for combination therapy with CT agents is still in a state of ambiguity [12,13,30]. Hence, this investigation evaluated the efficiency of the combination of CT and CP for cancer treatment.

Through clustering the results of text mining, it was revealed that the effects of CT agents were different from the impacts of CP agents on cancer treatment. The results indicated that CT agents, with their many unfavorable influences, complicate the cancer treatment process by developing cancer cell resistance [31–33]. There was at least one positive cluster (CT or CP) in 95% of the data acquired from the grouping of the text-mining results. Group 2 was the largest, indicating that CP agents can retrieve the disadvantages of CT agents in many cases. Whether CP agents, if combined with CT agents, could cover these disadvantages should be validated in practical experiments.

Since targeting all the disadvantages of CT agents to improve their performance is not easy, intelligent therapy targets the most significant ones. The most important hub genes of the four groups were extracted by measuring six important centralities of the PPI network. These hub genes, which are engaged in various cellular processes, were the most effective genes obtained from PPI analysis in the human genome network. Introducing these genes can pave the way for intelligent therapy in combination with cancer therapy. Interestingly, the results of a study by Notarbartolo *et al*. in 2005 were similar to the findings of the present research in relation to the hub genes in the PPI of Group 2 [34]. In Notarbartolo *et al*.’s study, curcumin reduced the increased NF-κB levels of doxorubicin treatment in HA22T/VGH hepatic cancer cells. Another study showed that curcumin reduced NF-κB (p56) delivery to the nucleus, thereby reducing the enhanced effect of NF-κB by doxorubicin [35].

The enrichment results indicated that the Group 1 and Group 2 hub genes were involved in various processes closely related to cancer and its treatment. As one of the main programmed cell death processes, apoptosis was one of the most important enriched processes for Group 1. Among enriched processes of Group 2, NF-κB activity, angiogenesis, inflammation, apoptosis, and cell cycle were the most important ones, all of which play vital roles in cell fate and resistance to treatment. These findings illustrated that CP agents, in combination with CT agents, can be effective in crucial processes related to cancer treatment.

The hub genes obtained from text mining and PPI of combined Hst and VCR were examined in the MCF-7 cancer cell line to validate the *in silico* results (Fig 4). The cell viability assay revealed that in most of the doses used, the lethality of VCR combined with Hst was cumulative and no antagonistic or synergistic effect was observed. Although Hst did not have a synergistic effect on cell death, its additive effect could be beneficial for combination therapy with CT agents. It has been reported that CP agents cause a synergistic or additive effect on cancer cell death [36–39].

*In silico* analysis results were confirmed by western blot and real-time PCR. The NF-κB protein is one of the most important proteins that determines cell fate and it is also involved in drug resistance [40,41]. Text-mining results illustrated that the expression level of this gene was increased by CT agents, which could lead to cell growth, proliferation, and treatment resistance [42]. In addition, this gene was nominated as one of the most important hub genes in the PPI analysis. Interestingly, Hst reduced the expression of NF-κB in the combined dose and prevented the effect of VCR on the elevated expression of this protein. This effect may cause cancer cells to be more susceptible to apoptosis and reduce resistance to treatment.

Apoptosis is one of the main therapeutic strategies to fight cancer. Caspase 3 cleavage is known as a fundamental apoptosis marker [43]. Martino *et al*. (2018) reported that VCR, in combination with other drugs, induced cell death more efficiently [44]. Similarly, the combination of VCR with *Satureja khuzestanica* extract induced apoptosis in MCF-7 cancer cells [45]. Therefore, it can be concluded that combined doses can synergistically induce apoptosis, which was supported by results of western blotting, indicating proper compatibility of these two agents for apoptosis induction. Chen *et al*. (2002) demonstrated that reducing NF-κB and BCL-2 expression induced apoptosis in the human clone cell [46]. Thus, this synergistic effect on the induction of apoptosis may be due to the inhibitory effect of Hst on the *NF-κB* expression. The analysis of the *TP53*, *MYC*, *BCL-2*, and *JUN* genes performed by real-time PCR also supported the appropriate compatibility of these two agents for cancer therapy which were consistent with the *in silico* analysis. The *TP53* gene, the most important cell guard, showed an increase in expression under the influence of different doses and the combined dose. This increase in expression can be a switch for inducing apoptosis [47]. The *BCL-2* and *MYC* genes, important genes for resistance to apoptosis [48,49], indicated a decrease under various treatments. The *JUN*, one of the genes stimulated by NF-κB [50], showed a decrease in the group treated with Hst but it did not change significantly in treatment with VCR. Both *NF-κB* and *JUN* genes were reduced in the combination group, which could cause a reduction in cell growth and induction of apoptosis.

Although the *in vitro* results confirmed the *in silico* results, for a better understanding of molecular mechanisms involved in apoptosis induced by combining drug agents, more comprehensive studies such as whole-cell transcriptome analysis are required. In some studies, the combination of CP and CT effects has not shown satisfactory results against cancer [14,15] when oral and non-targeted administration were used during chemotherapy. Thus, it is suggested that new methods of intelligent drug delivery should be used in addition to targeting the hub genes obtained in this study to achieve the effective performance of combination therapy with these two types of agents. For example, a study illustrated that nanoparticles loaded with docetaxel and resveratrol made HER-2-positive breast cancer cells sensitive to docetaxel [51].

## Conclusion

In this study, an integrated text mining and PPI analysis approach was used for the first time to investigate the combined effects of therapeutic agents for cancer treatment. The results revealed that CP agents could eliminate many CT disadvantages in many cancer treatment processes. The enrichment analysis of these disadvantages indicated that they were involved in essential and effective processes in cancer treatment. For instance, some of these crucial processes were TNF signaling pathway, the NF-κB signaling pathway, inflammatory response, etc. Some hub genes of this study can be a therapeutic target for intelligent cancer treatment with these agents. It was also illustrated that the effects of VCR in combination with Hst had a good harmony in cancer treatment which could partly validate the results of *in silico* analysis.

## Supporting information

S1 Fig Graph of human protein-protein interaction network. (PDF)

S2 Fig Text-mining clustering based on the impact of each agent on the cancer treatment process. (PDF)

S3 Fig Hub-genes of top 10 centralities obtained from PPI analysis. (PDF)

S1 File Text-mining results for cancer treatment. (XLSX)

S1 Data (XLSX)

S2 Data Enrichment analysis for Hub-genes derived from PPI. (XLSX)

S1 Raw images (PDF)

## Abbreviations

VCR: vincristine
Hst: hesperetin
PPI: protein-protein interaction
CT: chemotherapeutic
CP: chemopreventive
