# Supplementary figures and images for: The use of integrated text mining and protein-protein interaction approach to evaluate the effects of combined chemotherapeutic and chemopreventive agents in cancer therapy
Source: PLoS One. 2022 Nov 11;17(11):e0276458. doi: 10.1371/journal.pone.0276458 (PMC9651583; doi:10.1371/journal.pone.0276458)

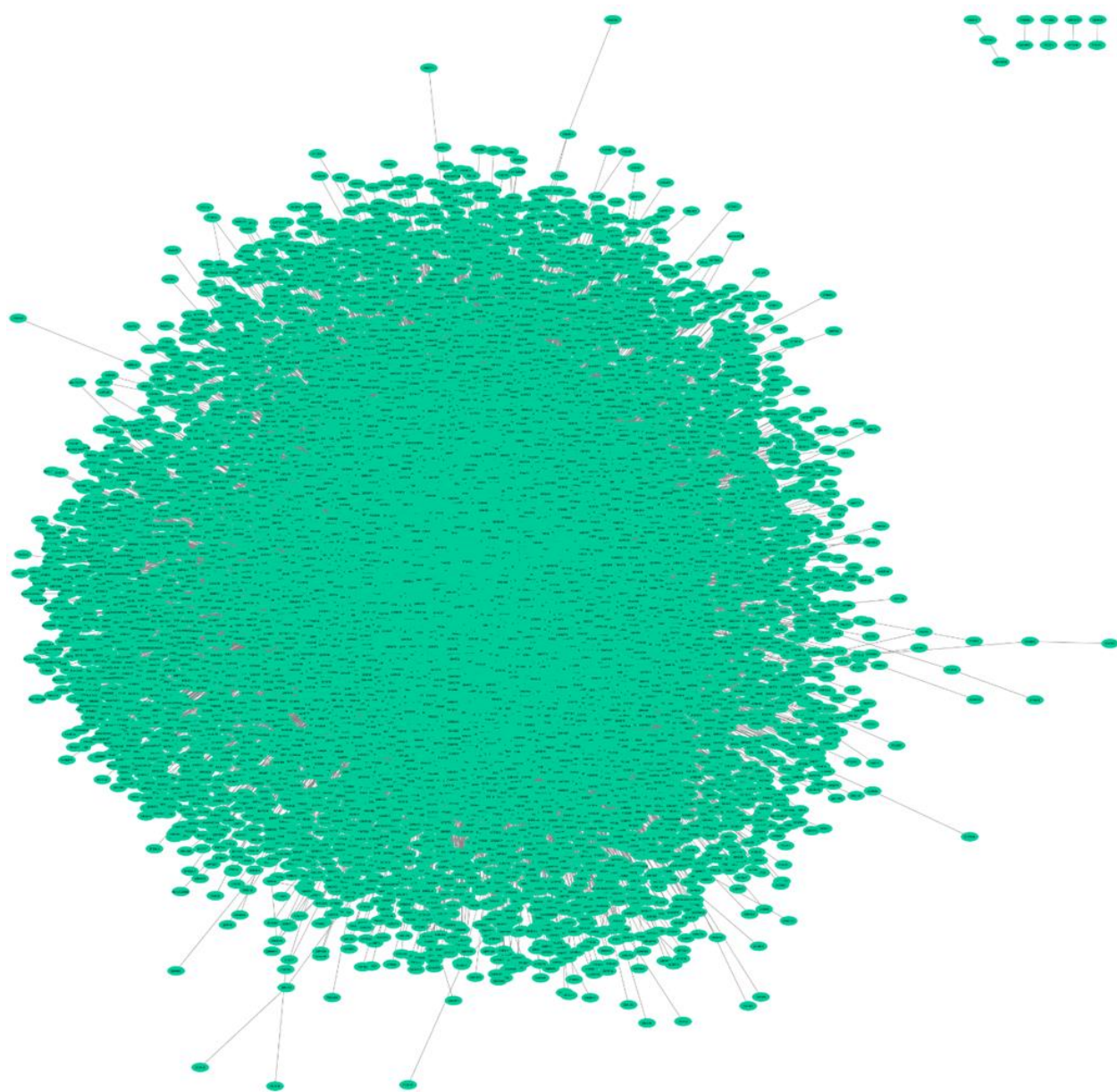

**S2 Fig. Graph of human protein-protein interaction network.**

Supplement: S1 Fig — (PDF) [file pone.0276458.s001.pdf]

A

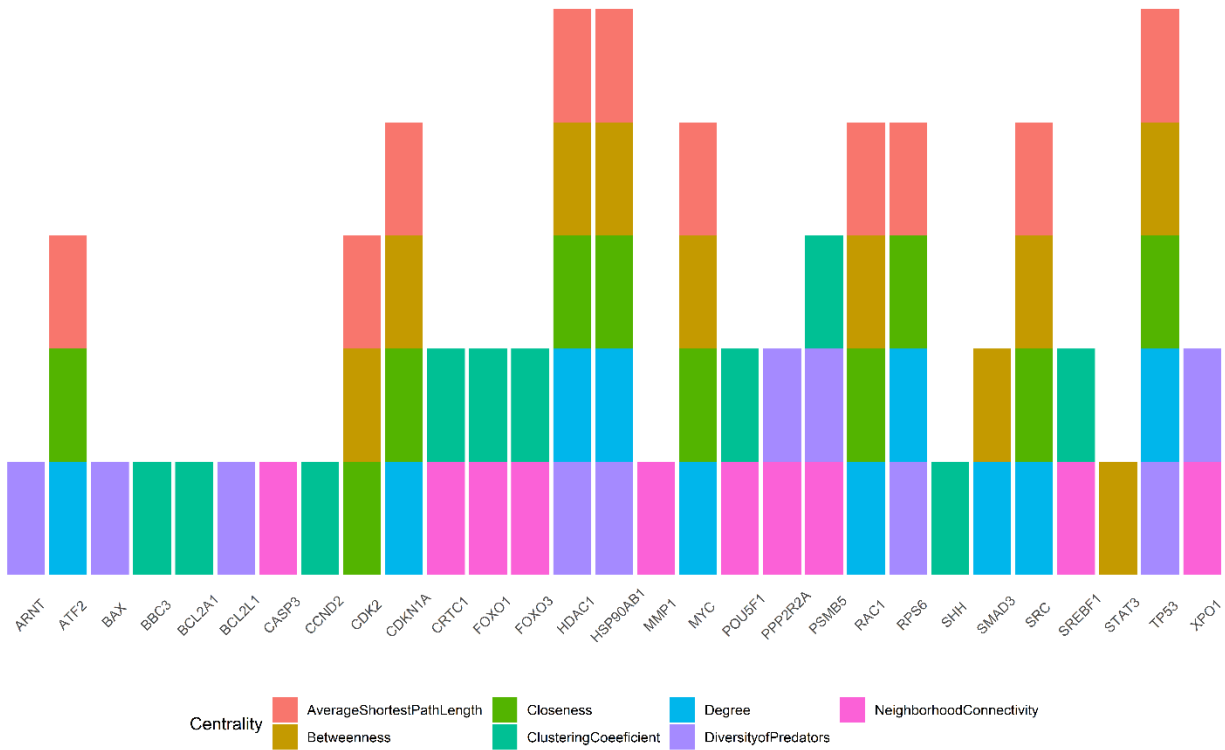

B

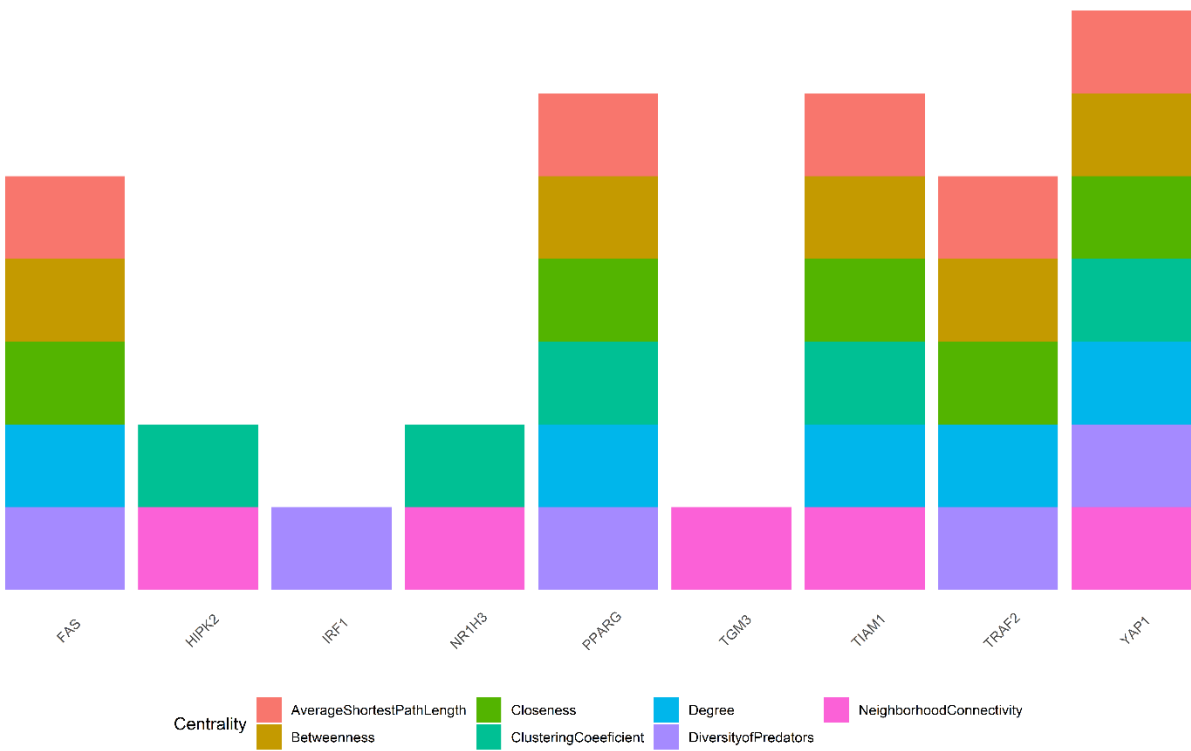

C

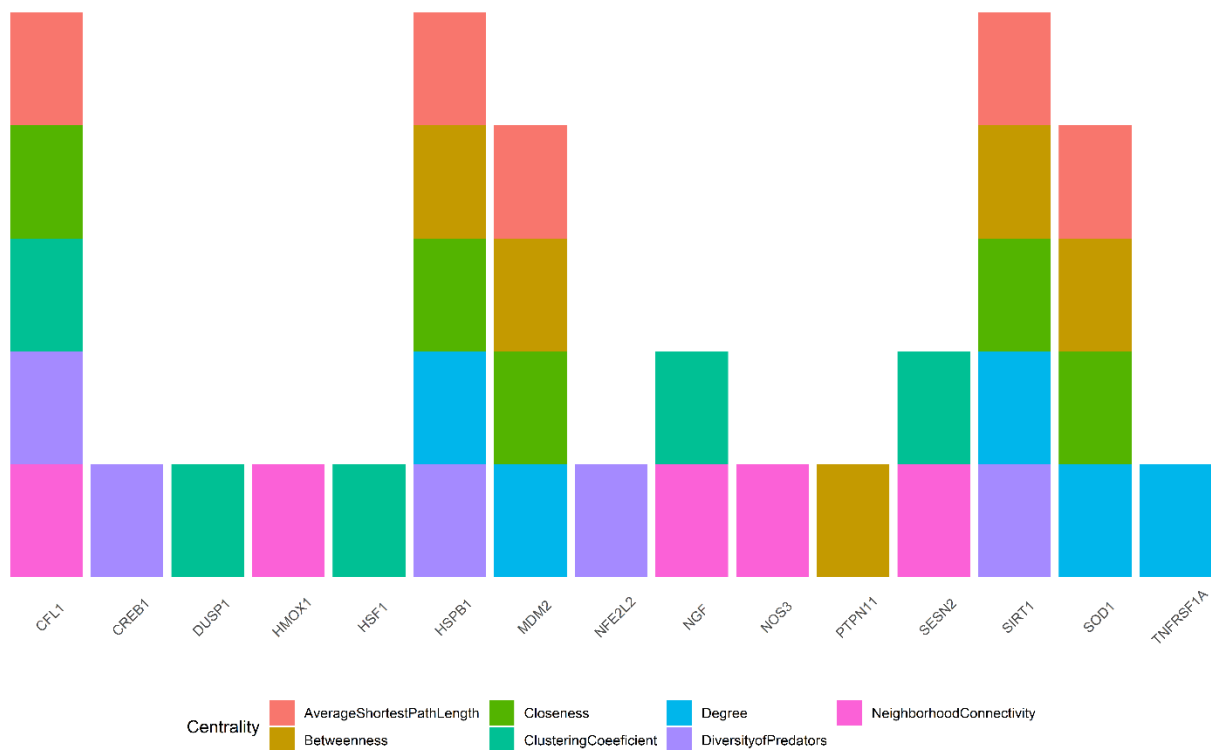

**S4 Fig. Hub-genes of top 10 centrality obtained from PPI analysis. A: group1. B: group3. C: group 4.**

Supplement: S3 Fig — (PDF) [file pone.0276458.s003.pdf]

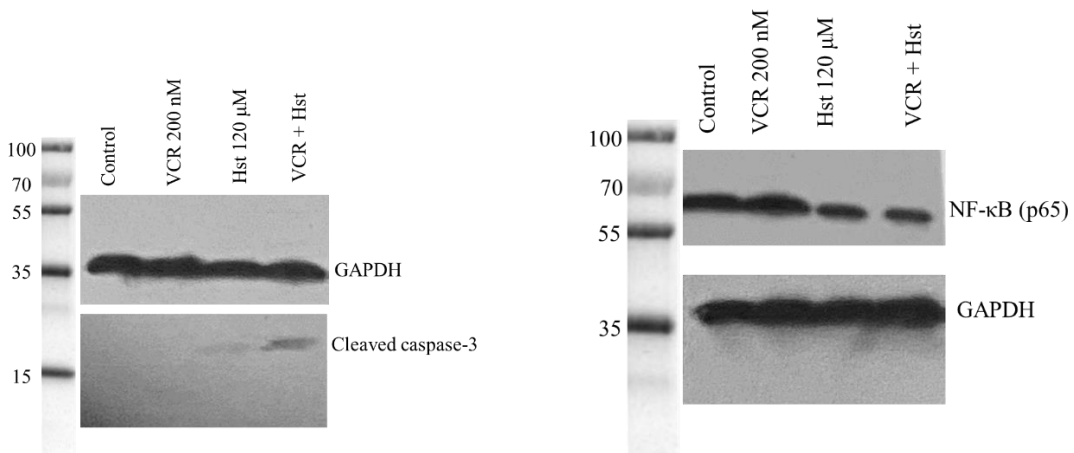

1- Western blotting lanes with ladder

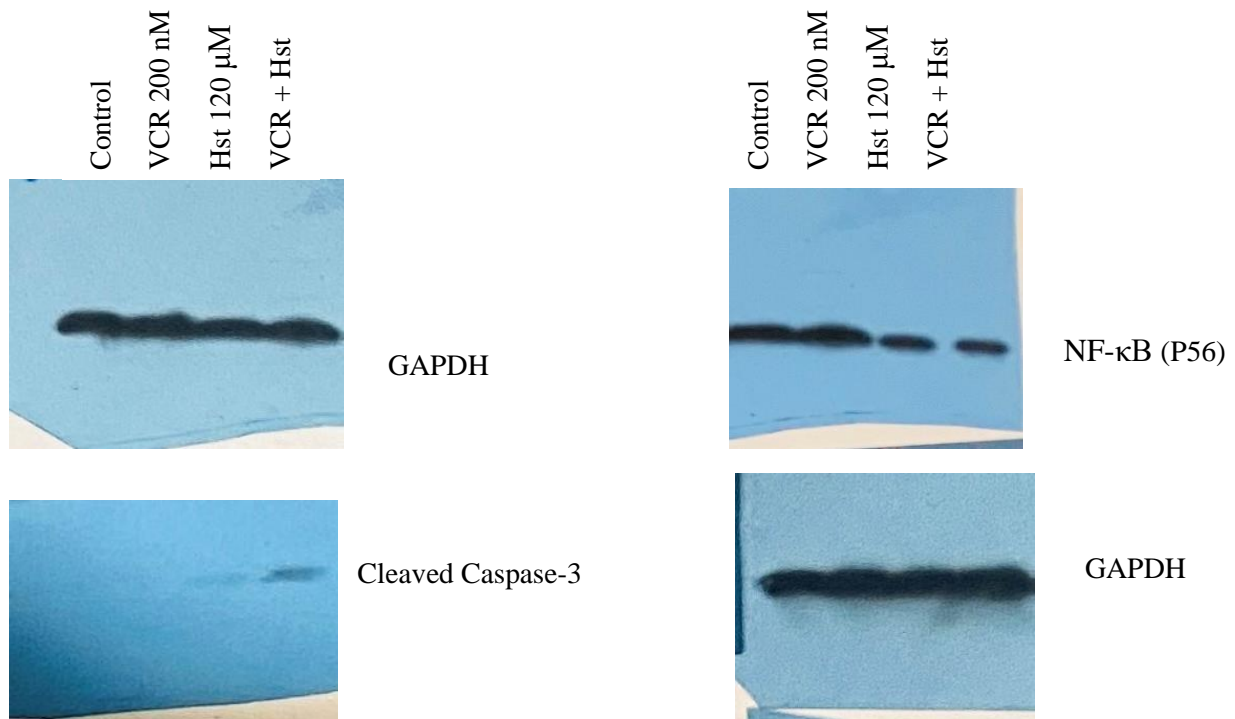

2- Original western blotting films

Supplement: S1 Raw images — (PDF) [file pone.0276458.s007.pdf]
