# Supplementary material for: The use of integrated text mining and protein-protein interaction approach to evaluate the effects of combined chemotherapeutic and chemopreventive agents in cancer therapy
Source: PLoS One. 2022 Nov 11;17(11):e0276458. doi: 10.1371/journal.pone.0276458 (PMC9651583; doi:10.1371/journal.pone.0276458)
Supplement: S2 Fig — (PDF) [file pone.0276458.s002.pdf]

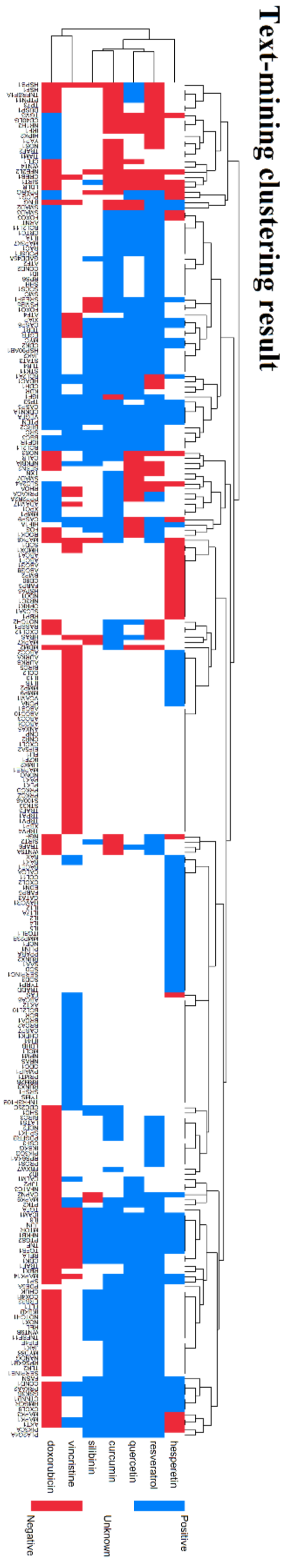

S3 Fig. Text-mining clustering based on the impact of each agent on the cancer treatment process.
